# Monitoring Autophagy in Rice With GFP-ATG8 Marker Lines

**DOI:** 10.3389/fpls.2022.866367

**Published:** 2022-04-25

**Authors:** Rui Liu, Rongxue Zhang, Yi Yang, Xuejun Liu, Qingqiu Gong

**Affiliations:** ^1^State Key Laboratory of Microbial Metabolism, Joint International Research Laboratory of Metabolic and Developmental Sciences, School of Life Sciences and Biotechnology, Shanghai Jiao Tong University, Shanghai, China; ^2^Tianjin Key Laboratory of Crop Genetics and Breeding, Tianjin Agricultural University, Tianjin, China; ^3^College of Life Sciences, Nankai University, Tianjin, China

**Keywords:** autophagy, ATG8, rice, autophagic flux, post-transcriptional regulation

## Abstract

Autophagy is a conserved intracellular trafficking pathway for bulk degradation and recycling of cellular components in eukaryotes. The hallmark of autophagy is the formation of double-membraned vesicles termed autophagosomes, which selectively or non-selectively pack up various macromolecules and organelles and deliver these cargoes into the vacuole/lysosome. Like all other membrane trafficking pathways, the observation of autophagy is largely dependent on marker lines. ATG8/LC3 is the only autophagy-related (ATG) protein that, through a covalent bond to phosphatidylethanolamine (PE), associates tightly with the isolation membrane/pre-autophagosomal structure (PAS), the growing phagophore, the mature autophagosome, and the autophagic bodies. Therefore, fluorescent protein (FP)-tagged ATG8 had been widely used for monitoring autophagosome formation and autophagic flux. In rice (*Oryza sativa*), FP-OsATG8 driven by Cauliflower mosaic virus (CaMV) 35S promoter had been used for imaging autophagosome and autophagic bodies. Here, we constructed three vectors carrying *GFP-OsATG8a*, driven by *35S*, *ubiquitin*, and the endogenous *ATG8a* promoter, individually. Then, we compared them for their suitability in monitoring autophagy, by observing GFP-ATG8a puncta formation in transiently transformed rice protoplasts, and by tracking the autophagic flux with GFP-ATG8 cleavage assay in rice stable transgenic lines. GFP-Trap immunoprecipitation and mass spectrometry were also performed with the three marker lines to show that they can be used reliably for proteomic studies. We found out that the ubiquitin promoter is the best for protoplast imaging. Transgenic rice seedlings of the three marker lines showed comparable performance in autophagic flux measurement using the GFP-ATG8 cleavage assay. Surprisingly, the levels of GFP-ATG8a transcripts and protein contents were similar in all marker lines, indicating post-transcriptional regulation of the transgene expression by a yet unknown mechanism. These marker lines can serve as useful tools for autophagy studies in rice.

## Introduction

Plants are constantly in need of nutrient reallocation during growth and development, yet they are continuously challenged by nutrient limitation and stresses. To combat starvation, biotic, and abiotic stresses while maintaining growth, plants have to efficiently remobilize and reallocate nutrients and clear up pathogens, damaged proteins, and even organelles. Among the degradation/remobilization pathways employed by plants, an intracellular trafficking pathway termed autophagy is particularly important, and the defects in autophagy strongly compromise biomass production and yield (Li et al., 2015; Wada et al., 2015; Have et al., 2017; Avin-Wittenberg et al., 2018; Signorelli et al., 2019; Cao et al., 2020; McLoughlin et al., 2020; Su et al., 2020).

Autophagy is an evolutionarily conserved, bulk degradation pathway of eukaryotic cells that can eliminate and recycle damaged or obsolete proteins and organelles (Nakatogawa, 2020; Zhang et al., 2021). In this pathway, the cargoes, either recognized selectively or non-selectively (Floyd et al., 2012; Farre and Subramani, 2016), are firstly sequestered to a double-membraned vesicle termed isolation membrane (IM) or phagophore, at a specific site termed phagophore assembly site (PAS) usually close by the endoplasmic reticulum (ER; Hollenstein and Kraft, 2020; Wun et al., 2020). Then, the phagophore expands and eventually seals to form an autophagosome. The autophagosome fuses with the tonoplast, and the cargoes, together with the inner membrane (termed autophagic bodies), are released into the vacuole for degradation (Yim and Mizushima, 2020). Through transporters yet unidentified in plants, the amino acids and other macromolecules are transported back into the cytoplasm. Clearly, to monitor the entire process, a marker protein that labels the isolation membrane, autophagosome, and autophagic bodies is essential. ATG8 is the protein (Contento et al., 2005).

ATG8 of yeasts and plants, and LC3/GABARAP of animals, was firstly discovered in the budding yeast (*Saccharomyces cerevisiae*), through the screen for autophagy (as APG8; Tsukada and Ohsumi, 1993), cytoplasm to vacuole targeting (Cvt; as CVT5; Harding et al., 1995), and pexophagy (as AUT7) mutants (Harding et al., 1996). ATG8 is an ubiquitin-like (Ubl) protein that scaffolds the expanding phagophore and autophagosome (Nakatogawa et al., 2007; Xie et al., 2008). After processing by the cysteine protease ATG4, a C-terminal glycine residue of ATG8 is exposed and gets conjugated to the lipid phosphatidylethanolamine (PE) by the ATG8 conjugation system composed of ATG7 as the E1 activating enzyme, ATG3 as the E2 conjugating enzyme, and ATG12-ATG5 complex as the E3 ligase (Mizushima et al., 1998). PE-conjugated ATG8 stays on the expanding phagophore and the mature autophagosome. Then, ATG4 cleaves PE-conjugated ATG8 off the outer membrane of autophagosome before it fuses with the vacuole (Yu et al., 2012). Since it is attached to both sides of the phagophore, upon autophagosome closure, approximately one-third of ATG8 molecules are trapped on the inner membrane of autophagosome along with the cargoes (Nair et al., 2012), thus gets degraded in the vacuole. The fact that ATG8 cannot be fully recycled before autophagosome-vacuole fusion may explain why it is strongly transcriptionally upregulated by autophagy-inducing conditions (Yoshimoto et al., 2004; Nair et al., 2012).

ATG8 not only controls phagophore expansion but is a key element in cargo sequestration. Using their ATG8-interacting motif (AIM)/LC3-interacting region (LIR), ULK1/ATG1 and ATG13 bind ATG8 to regulate autophagosome formation (Wild et al., 2014). Autophagy receptors, adaptors, and cargoes bind ATG8 with AIM/LIR or the ubiquitin-interacting motif (UIM) to get packed into the expanding phagophore (Marshall et al., 2019; Johansen and Lamark, 2020). Possibly for this reason, ATG8 has become arguably the most frequently studied ATG protein. Screens that designed to identify new players in autophagy generally centered on ATG8 (Honig et al., 2012; Zeng et al., 2021). In the autophagy protein–protein interaction network, ATG8 is always a node (Behrends et al., 2010; Tu et al., 2021).

Apart from the shared characteristics with the yeast and animal ATG8s, the plant ATG8s have unique properties (Bu et al., 2020). Arabidopsis has nine ATG8s (ATG8a-i; Yoshimoto et al., 2004), maize has five, and rice has seven that can be mapped (Chung et al., 2009; Xia et al., 2011). The plant ATG8s are categorized into three sub-families and the expansion of the ATG8 family was thought to take place early in the green lineage (Kellner et al., 2017; Zhang et al., 2021). Transcriptome data gave diverse expression patterns of ATG8s of Arabidopsis, indicative of their tissue specific roles (Thompson et al., 2005). Master regulator that governs autophagosome and lysosome biogenesis, such as transcription factor EB (TFEB; Settembre et al., 2011), has not been identified in plants, yet searches for plant-specific transcription regulators led to the discovery of TGACG (TGA) motif-binding protein 9 (TGA9) as a positive regulator of autophagy (Wang et al., 2020). ELONGATED HYPOCOTYL 5 (HY5), along with HISTONE DEACETYLASE 9 (HDA9), was discovered to repress the transcription of autophagy genes such as ATG8e and ATG5 during light-to-dark transition (Yang et al., 2020), thus providing an answer for an open question in plant autophagy.

The way ATG8 is used as a marker for autophagy is also unique in plants. Firstly, the fact that plants have many ATG8s with different molecular weights prevented us from using one anti-ATG8 antibody to examine the autophagic flux, for it is futile to separate different ATG8 isoforms from the PE-conjugated ATG8s. Secondly, the plant vacuole (pH 5.4–5.8) is not as acidic as the lysosome (pH 4.5); hence, the acid-sensitive GFP tag is not promptly degraded in the vacuole. For this reason, it is not possible to use a double tagged ATG8, such as mCherry-GFP-LC3, and take the fluorescent color change as an indicator for autophagic flux (Kaizuka et al., 2016). So far, the relatively reliable way is to use FP-ATG8 transgenic lines and a FP antibody, and to treat the plants with or without V-ATPase inhibitors, such as Concanamycin A (ConA), to distinguish the free FP band (presumably residing in the lytic vacuole as a consequence of autophagy, more resistant to vacuolar hydrolases than ATG8 due to its structural feature) from the FP-ATG8 band (presumably outside the vacuole). This method is termed FP-ATG8 cleavage assay. Selection of a proper promoter for the GFP-ATG8 transgenic line is also important, for ectopic expression of ATG8 often promotes transition into flowering, improves nitrogen use efficiency, and increases yield (Chen et al., 2019; Yu et al., 2019), and thus could be unsuitable for stress and developmental studies.

For autophagy studies in rice, *35S:mRFP-OsATG8a* and *35S:mRFP-OsATG8d* lines were firstly generated and imaged for autophagosome accumulation upon ConA treatments (Izumi et al., 2015). Recently, *35S:GFP-OsATG8a* and *35S:GFP-OsATG8b* lines have been generated, and both were reported to increase yield (Yu et al., 2019; Fan et al., 2020). The lack of a comparison between different *GFP-OsATG8* constructs prompted us to generate OsATG8 monitoring lines with *35S*, *ubiquitin*, and endogenous promoters, and to compare their performance under autophagy-inducing conditions. We aimed to pin down specific constructs and lines that are suitable for documenting autophagy, *via* imaging or immunoblotting. We also wanted to evaluate the potential of GFP-OsATG8 in protein–protein interaction screen, which have been a powerful tool in the identification of new players in plant autophagy (Honig et al., 2012; Han et al., 2015; Zeng et al., 2021).

## Materials and Methods

### Plasmids Construction

In this study, the *pCAMBIA1302* vector was used for generating transgenic rice lines. The coding sequence of *OsATG8a* (*Os07g0512200*; 360 bp in length) was amplified from rice cDNA and inserted at the *Eco91*I site through homologous recombination as described (Luo et al., 2017). To generate *ProUBQ10:GFP-OsATG8a* and *ProATG8a:GFP-OsATG8a* plasmids, the *35S* promoter was replaced with the *ubiquitin* promoter and the *OsATG8a* promoter, respectively, and inserted between HindIII and NcoI sites. All constructs were verified by DNA sequencing. The primers used for cloning, plasmid construction, and sequencing are listed in Supplementary Table S1.

### Generation of Transgenic Rice Lines and Plant Growth Conditions

Transgenic rice lines were generated similarly to a previous report on OsATG8s (Izumi et al., 2015). Briefly, sterilized rice seeds (japonica rice cultivar Jinjing 818) were used for callus induction. The vectors were transformed into mature seed-derived rice calli by Agrobacterium-mediated transformation. Transgenic rice plants (T1) were obtained through several rounds of differentiation inductions and selected on medium containing Hygromycin B. These T1 transgenic rice lines were selected by liquid medium containing 50 mg/L Hygromycin B for 3 days. After germinating in plates containing water for 5–6 days, the seedlings were grown in the modified Hoagland’s solution (5 mM KNO_3_, 2 mM MgSO_4_·7H_2_O, 5 mM Ca(NO_3_)_2_·4H_2_O, 1 mM KH_2_PO_4_, 0.05 mM FeSO_4_·7H_2_O, 0.05 mM Na_2_EDTA, 46 nM H_2_BO_3_, 9 nM MnCl_2_·4H_2_O, 0.3 nM CuSO_4_·5H_2_O, and 0.8 nM ZnSO_4_·7H_2_O) at 28°C/24°C, 14 h light/10 h dark in a growth chamber. Transgenic rice was cultivated in paddy fields in growth seasons of 2019 to 2021 in Tianjin, Jiangsu, and Hainan provinces of China.

### Transient Transformation of Tobacco Leaves

*GFP-OsATG8a* driven by three different promoters were transiently expressed in tobacco leaves as described (Luo et al., 2017). Soil grown, 4-week-old *N. benthamiana* leaves were used for Agrobacterium infiltration. After 2 days of inoculation, leaves were collected and cut into small squares for confocal microscopy.

### Rice Protoplast Transformation

Sterilized rice seeds (japonica rice cultivar Jinjing 818) were germinated on 1/2 Murashige and Skoog (MS) medium with a photoperiod of 14 h light and 10 h dark at 26°C for 5–6 days, then moved to the dark for another 5–6 days. The etiolated stem and sheath of rice seedlings were cut into pieces of approximately 0.5 mm with sharp razors. These pieces were immediately transferred into 20 ml enzyme solution (1.5% Cellulase RS, 0.75% Macerozyme R-10, 0.6 M mannitol, 10 mM MES at pH 5.7, 10 mM CaCl_2_, and 0.1% BSA), vacuum infiltrated for 30 min, and further digested in the dark for 4–5 h with gentle shaking. After enzyme digestion, protoplasts were released by adding an equal volume of W5 solution (154 mM NaCl, 125 mM CaCl_2_, 5 mM KCl, and 2 mM MES at pH 5.7) and gentle shaking by hand for 2 min. Then, the protoplasts were filtered through a 40 μm-gauge nylon mesh with 3–5 washes using W5 solution and collected by spinning at 100 g for 3 min. After protoplasts were re-suspended and washed once with W5 solution, MMG solution (0.4 M mannitol, 15 mM MgCl_2_, and 4 mM MES at pH 5.7) was used to re-suspend the pellets at a concentration of 2 × 10^6^ cells ml^−1^. For each transformation, 6–8 μg of freshly prepared plasmid DNA and 200 μl protoplasts (about 4 × 10^5^ cells) were mixed with 220 μl freshly prepared PEG solution [40% (w/v) PEG 4000, 0.4 M mannitol, and 0.1 M CaCl_2_] and were incubated at room temperature for 30 min in the dark. After incubation, the protoplasts were mixed with 1 ml W5 solution and incubated at 28°C for 12–15 h in the dark before imaging.

### Laser Scanning Confocal Microscopy

Transiently transformed tobacco leaves (lower epidermis) and rice protoplasts were observed with a Ni-E A1 HD25 confocal microscope (Nikon, Japan). Prior to image collection, the background auto-fluorescence was eliminated using untransformed tobacco leaves or rice protoplasts. The GFP fluorescence signal was exited at 488 nm and emission was collected at 500–550 nm. The chlorophyll auto-fluorescence was exited with 561 nm laser and emission was collected at 600–700 nm. For NaCl treatments, protoplasts were incubated in W5 solution with 250 mM NaCl, or 50 μM E-64d, or both NaCl and E-64d, for 30 min at room temperature. For inhibitor treatments, transformed protoplasts were incubated in W5 solution containing 200 nM AZD8055, or 1 μM ConA, or both AZD8055 and ConA, for 3 h at room temperature.

### Quantitative Real-Time RT-PCR

Leaves from 14-day-old seedlings were used for RNA extraction, cDNA synthesis, and quantitative real-time RT-PCR (qRT-PCR) as described (Luo et al., 2017). Transcript levels of *GFP* and *OsATG8a* were normalized to *OsEF1a (LOC_Os03g08020)* with three biological replicates consisting of four technical repeats. Specific amplification was verified by a melt curve analysis following the completion of the PCR cycles. Each PCR product generated a single peak in melt curve analysis, indicating specific amplification. The 2^-ΔΔCT^ method was used for relative quantification of qRT-PCR data. Primers used are listed in Supplementary Table S1.

### Immunoblotting

Two-week-old seedlings were used for autophagic flux measurement. Excised leaves were incubated in the Hoagland’s solution with 0.01% Tween-20, and stirred and vacuumed to make sure the leaves were completely soaked in the solution. For NaCl treatments, excised leaves were incubated in the Hoagland’s solution plus 150 mM NaCl, or 100 μM E-64d, or both NaCl and E-64d, for 1 h at room temperature. Alternatively, 250 mM NaCl was used. For inhibitor treatments, excised leaves were incubated in the Hoagland’s solution containing 2 μM AZD8055, or 1 μM ConA, or both AZD8055 and ConA, for 6 h at room temperature. In addition, 6 h with 200 μM BTH plus 100 μM E-64d treatment and 4 h with 2 mM DTT plus 100 μM E-64d treatment were used to induce autophagy. Protein extraction and immunoblotting were done as described (Luo et al., 2017). Semi-quantification of the protein levels was performed with ImageJ^1^ and protein levels were normalized to Coomassie brilliant blue R-250-stained band of the RuBisCO large subunit (RbcL). For immunoblotting, mouse anti-GFP (1:5,000 dilution, Utibody, China), rabbit anti-H3 (1:8,000 dilution, ABclonal, China), and the appropriate IgG-HRP conjugated secondary antibody (1:5,000; ZSGB-Bio, China) were used. The signal was developed using High-sensitivity ECL chemiluminescence detection kit (Vazyme, China) and chemiluminescence was detected using a chemiluminescent Western Blot scanner (ChemiScope 6100T, Clinx, China). All experiments were repeated for three to five times, and one representative result was shown.

### GFP-Trap and Mass Spectrometry

Transgenic rice seedlings were frozen and ground in liquid nitrogen, and protein extraction buffer (50 mM Tris–HCl, pH 7.5, 150 mM NaCl, 20% Glycerol, 1 mM MgCl_2_, 0.2% NP-40, and 1% Protease Inhibitor Cocktail) was added at a proportion of 1:2 (m:v). Extracts were centrifuged at 12,000 rpm for 15 min at 4°C. Then, the supernatants were collected and centrifuged again at 12,000 rpm for 5 min at 4°C. The supernatants (1 ml to 10 μl bead volume) were incubated with GFP-Trap A beads pre-equilibrated in wash buffer (50 mM Tris–HCl, pH 7.5, 150 mM NaCl, 20% Glycerol, 1 mM MgCl_2_, and 0.1% NP-40) at 4°C for 2–3 h. The beads were washed 3–4 times with wash buffer and were re-suspended in 100 μl wash buffer. Samples were verified by Western blotting; the corresponding gels were cut and sent for mass spectrometry analysis at the Instrumental Analysis Center of Shanghai Jiao Tong University.

## Results

### The Ubiquitin Promoter Is More Suitable for the Observation of Transiently Expressed *GFP-OsATG8a*

**Figure 1:**
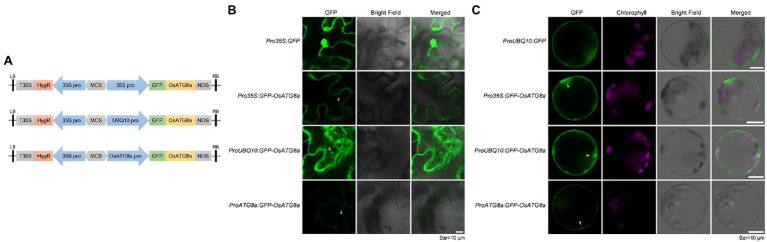
Transient expression of constructs carrying *GFP-OsATG8a*. **(A)** Schematic representation of the *GFP-OsATG8a* fusion gene constructs. Three promoters—the *CaMV35S* promoter, the *ubiquitin* (*UBQ10*) promoter, and the endogenous (*OsATG8a*) promoter—are used for the constructs. **(B)** LSCM of tobacco leaf epidermal cells transiently expressing GFP and GFP-OsATG8a. **(C)** Transient expression of GFP and GFP-OsATG8a in rice protoplasts. Puncta representing autophagosomes are indicated by yellow arrowheads in **(B,C)**. Bar = 10 μm in **(B,C)**.

We cloned OsATG8s from an herbicide-resistant japonica rice cultivar Jinjing 818 and examined their transcript levels. Consistent with previous reports (Xia et al., 2011; Izumi et al., 2015), OsATG8a was the most highly expressed isoform. Hence, it was selected for vector construction (Figure 1A). OsATG8d was also selected (Supplementary Figure S2); however, we were unable to obtain transgenic lines carrying *ProATG8d:GFP-OsATG8d*, and it was eventually left out.

Expression of *GFP-OsATG8a* driven by *35S*, *ubiquitin*, and *OsATG8a* (endogenous) promoters was firstly examined in transiently transformed *N. benthamiana* leaf epidermal cells. *Pro35S:GFP* gave typical, strong nuclear, and cytoplasmic GFP signals. *Pro35S:GFP-OsATG8a* gave relatively weak cytoplasmic signals and punctate signals that likely represent autophagosomes. Strong cytoplasmic/endoplasmic reticulum and punctate signals were observed from cells transformed with *ProUBQ10:GFP-OsATG8a* (Figure 1B)*. ProATG8a:GFP-OsATG8a* had the lowest expression level, with puncta representing autophagosomes observed (Figure 1B).

We then examined the expression levels of the three constructs in transiently transformed rice protoplasts (Figure 1C). Again, *ProUBQ10:GFP* gave cytoplasmic signals, whereas the *GFP-ATG8a* constructs gave both cytoplasmic and punctate signals. Similar to our observation in tobacco leaves, *ProUBQ10:GFP-ATG8a* gave strong and clear signals, and signal from *GFP-ATG8a* driven by the endogenous promoter was very weak.

**Figure 2:**
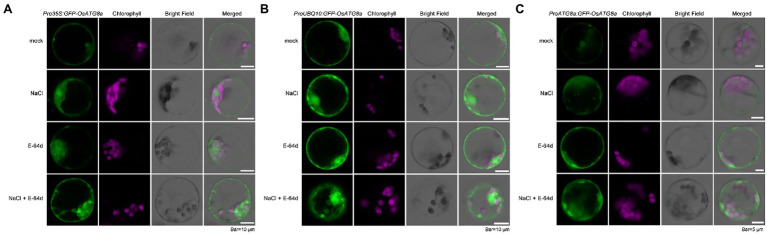
Visualization of autophagic body accumulation in rice protoplasts. Rice protoplasts expressing *GFP-OsATG8a* driven by **(A)** *35S* promoter, **(B)** *UBQ10* promoter, and **(C)** *OsATG8a* promoter were observed with LSCM after treatment with 250 mM NaCl, or 50 μM E-64d, or NaCl plus E-64d for 30 min. Accumulation of GFP signals, indicative of autophagosome and autophagic bodies, can be seen in protoplasts treated with NaCl plus E-64d. Bar = 10 μm in **(A,B)**; 5 μm in **(C)**.

We had previously shown that the autophagic flux can be induced by salt stress within 30 min in Arabidopsis (Luo et al., 2017). Here, we also treated the transformed rice protoplasts with 250 mM NaCl in the presence of a lysosomal cysteine protease inhibitor E-64d to see if autophagic flux could be induced by salt treatment (Figure 2). GFP-OsATG8a driven by all three promoters responded to salt treatment, as NaCl plus E-64d significantly induced GFP-OsATG8a accumulation (Figures 2A–C).

Since inhibition of Target of Rapamycin (TOR) can reliably induce autophagy, we treated the protoplasts with 200 nM AZD8055, a potent TOR inhibitor (Chresta et al., 2010), in the presence of the V-ATPase inhibitor ConA (Huss et al., 2002), to further validate the constructs. *GFP-OsATG8a* driven by *UBQ10* and *ATG8a* promoters both responded to TOR inhibition (Supplementary Figure S1).

Inferring from the expression intensities, the ubiquitin promoter is a good choice for the transient expression of OsATG8a.

### Stable Transgenic Lines Carrying *GFP-OsATG8a* Are Comparable for Immunoblotting

After validating and comparing the three constructs in transient expression systems, transgenic rice carrying these constructs were generated. T1 transgenic lines regenerated from the transformed calli were firstly validated with genomic PCR and immunoblotting (Supplementary Figures S2A,B). Then, the plants were grown in the paddy fields and measured for their heights and the number of tillers before harvesting. Consistent with a recent report (Fan et al., 2020), the transgenic lines were not very different among themselves in their heights (Supplementary Figure S3A). The numbers of tillers were not very different either (Supplementary Figure S3B). No significant change in phenotype was observed at the seedling stage in the T3 transgenic lines (Supplementary Figure S3C).

**Figure 3:**
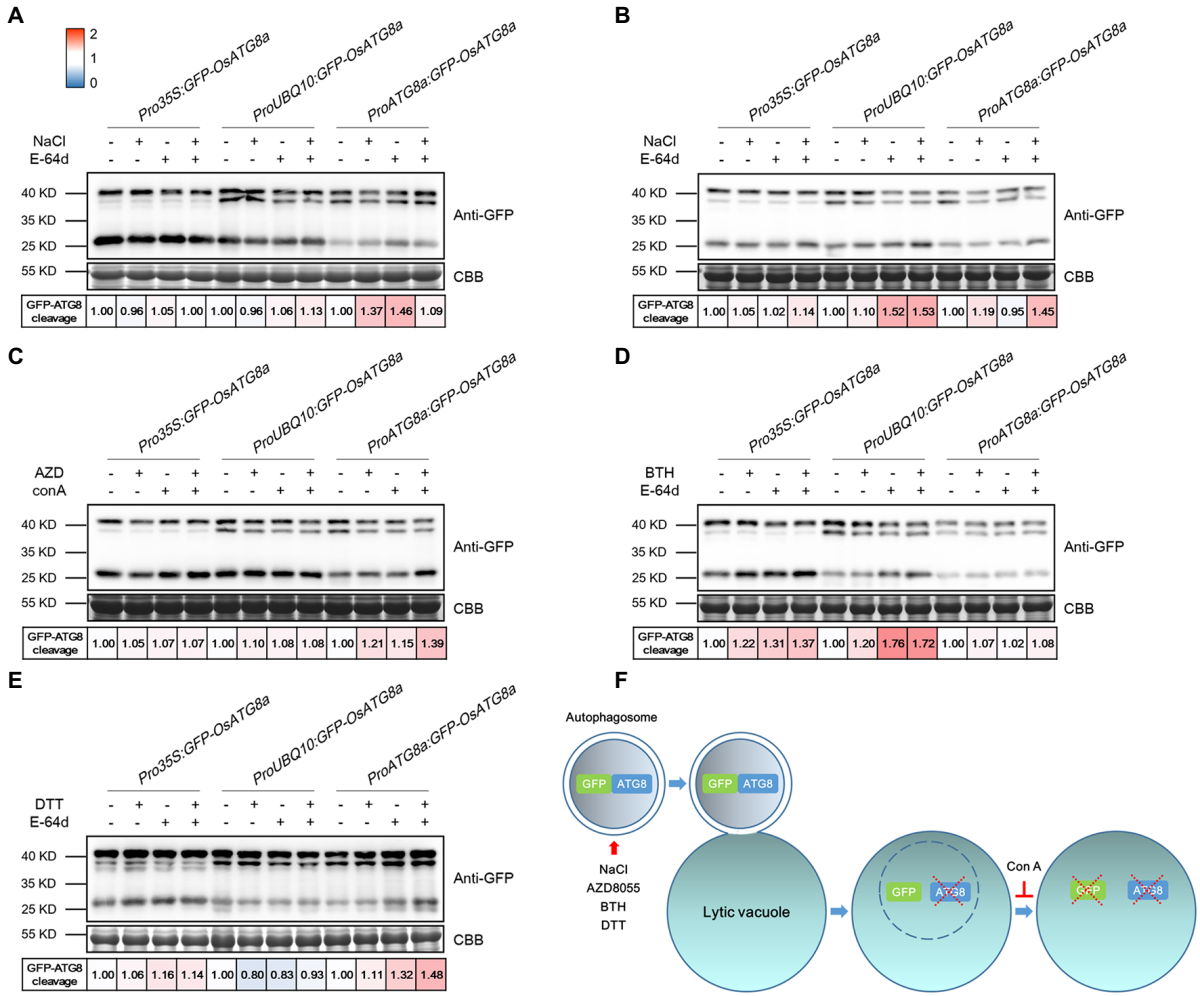
Measuring autophagic flux in *GFP-OsATG8a* marker lines. The level of autophagic flux is represented by the difference of the GFP-ATG8 cleavage between control and treatment. GFP-ATG8 cleavage was calculated as the ratio of the free GFP band (27 kD) to the total GFP signal in the lane (40kD plus 27 kD), then normalized to the respective control (no treatment, no E-64d or ConA; set as 1.00). A color scale (Blue for 0, white for 1, and red for 2) was used to illustrate the semi-quantified values of GFP-ATG8 cleavage. Coomassie Brilliant Blue (CBB) staining of RuBisCO large subunit was used as loading control. **(A)** For moderate salt stress-induced autophagy, excised leaves from 14-day-old rice seedlings were incubated in the Hoagland’s solution plus 150 mM NaCl, or 100 μM E-64d, or both NaCl and E-64d for 1 h. **(B)** For severe salt stress-induced autophagy, leaves were incubated in 250 mM NaCl and 100 μM E-64d for 1 h. **(C)** For TOR inhibition-induced autophagy, leaves were incubated in 2 μM AZD8055 and 1 μM Concanamycin A for 6 h. **(D)** For BTH-induced autophagy, 200 μM BTH and 100 μM E-64d for 6 h were used. **(E)** For ER stress-induced autophagy, 2 mM DTT and 100 μM E-64d for 4 h were used. At least three biological replicates were done for each treatment, and one representative replicate is shown. **(F)** A diagram illustrating the GFP cleavage assay and the chemicals used.

The T3 transgenic seedlings carrying *Pro35S:GFP-OsATG8a*, *ProUBQ10:GFP-OsATG8a*, and *ProOsATG8a:GFP-OsATG8a* were evaluated for their suitability in measuring autophagic flux. The autophagic flux was measured with the GFP-ATG8 cleavage assay, in which the flux is represented by the ratio of the amount of cleaved GFP (27 kD) to total GFP in the lane (40 kD plus 27 kD), i.e., GFP/(GFP + GFP-ATG8a). Firstly, GFP-OsATG8a (40kD) and free GFP (27kD) bands were readily detected in the transgenic lines (Figure 3). Since one or two bands were detected for GFP-OsATG8a on immunoblots, we validated the bands by running the samples along with recombinant GFP-OsATG8s purified from *E.coli*. Two bands of similar sizes were seen for the recombinant proteins GFP-OsATG8a and GFP-OsATG8d (Supplementary Figure S2C). Such observation indicated that both bands correspond to GFP-ATG8, and the amount of GFP-ATG8 should be measured as the sum of the two.

For autophagy induction, rice seedlings were treated with NaCl, AZD8055, BTH, and DTT (Figures 3A–E). Benzo-(1,2,3)-thiadiazole-7-carbothioic acid (BTH), a salicylic acid agonist, has been used to induce autophagy (Yoshimoto et al., 2009; Zeng et al., 2021). DTT is known to induce ER stress-elicited autophagy (Liu et al., 2012). In general, GFP-OsATG8a driven by all three promoters responded mildly to the treatments and chemicals. A possible explanation is that the basal level autophagy is relatively high already in rice. Specifically, *Pro35S:GFP-OsATG8a* responded relatively strongly to 200 μM BTH. *ProUBQ10:GFP-OsATG8a* responded well to 250 mM NaCl and BTH, but not to 2 mM DTT. *ProOsATG8a:GFP-OsATG8a* responded nicely to moderate and severe salt stress, AZD8055, and DTT. A diagram illustrating the GFP cleavage assay and the chemicals used is presented (Figure 3F). E-64d is omitted from the diagram because its precise role in preserving GFP, or even GFP-ATG8, is currently unknown.

**Figure 4:**
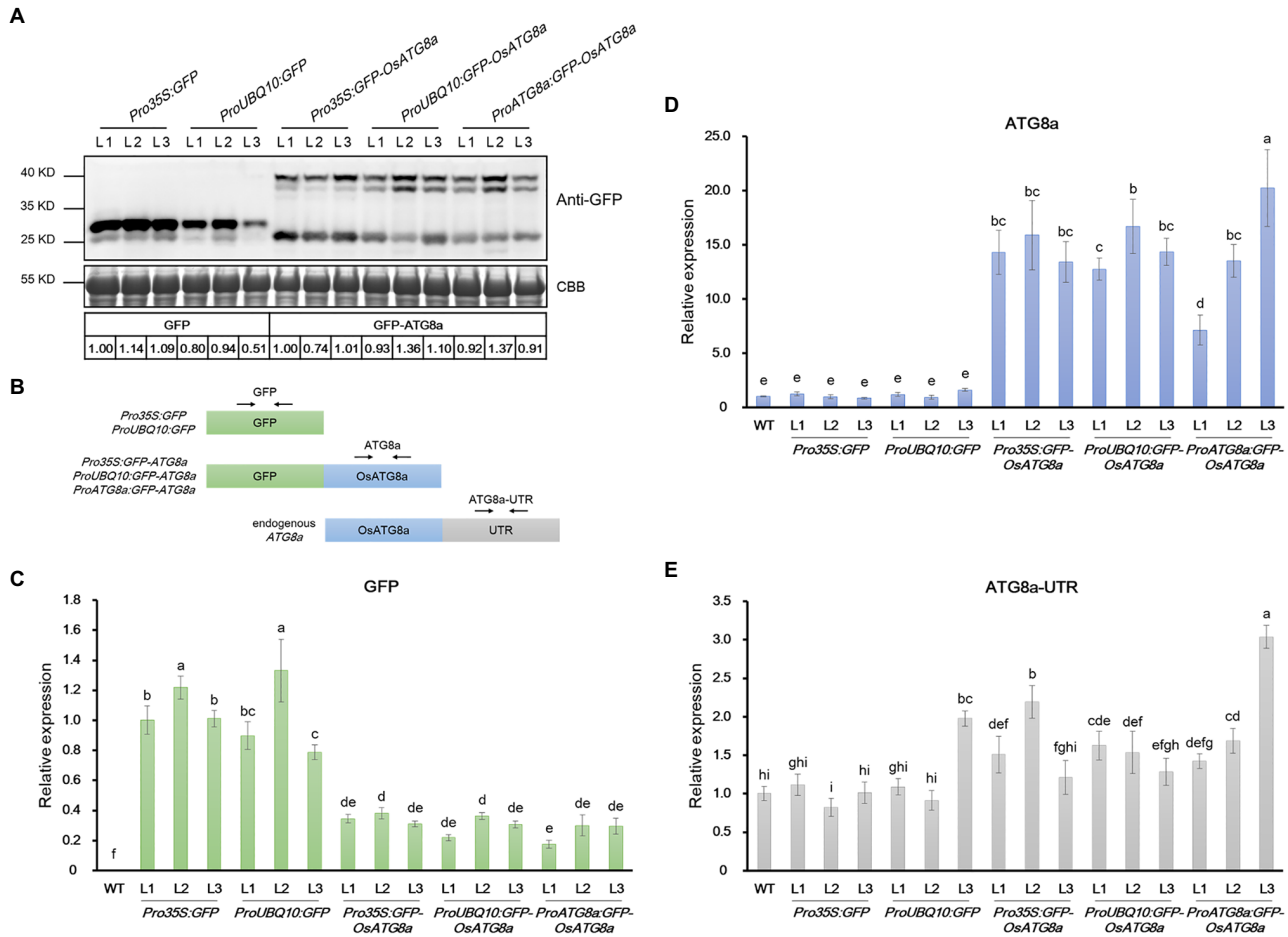
mRNA and protein levels of GFP and GFP-OsATG8a in rice transgenic lines. **(A)** Expression of GFP (27 kD) and GFP-OsATG8a (40 kD, one or two bands) were detected by immunoblotting and quantified by ImageJ. The protein level of GFP was normalized to *Pro35S:GFP*—L1 (set as 1), and the level of GFP-OsATG8a was normalized to *Pro35S:GFP-OsATG8a*—L1 (set as 1). Three T2 transgenic lines for each construct were shown. Coomassie Blue staining of RuBisCO large subunit was used as loading control. Three biological replicates were done, and one representative replicate is shown. **(B)** Schematic representation of the primers designed for qRT-PCR in **(C–E)**. **(C–E)** qRT-PCR analysis of transgenic seedlings. Transcript levels of **(C)** *GFP*, **(D)** *OsATG8a*, and **(E)** *OsATG8a-UTR* were profiled and normalized to the *Pro35S:GFP*—L1 **(C)** or the wild-type control **(D,E)**. Data are means ± SD (*n* = 4), one-way ANOVA followed by Tukey B test; *p* = 0.05. Different letters denote significant differences. Three biological replicates, each consisting of four technical repeats, were done, and one representative replicate is shown.

At this point, we noticed an interesting phenomenon. The T3 transgenic lines carrying *35S:GFP* and *Ubiquitin:GFP* gave strong GFP bands (Figure 4A; Supplementary Figure S2B), validating the strength of the promoters. However, lines carrying *GFP-OsATG8a* driven by *35S*, *ubiquitin*, or *OsATG8a* promoters had comparable protein levels of GFP-OsATG8a among themselves (Figure 4A, Supplementary Figure S2B). We then examined the transcript levels of the *GFP-OsATG8a* fusion gene and the endogenous *OsATG8a* with qRT-PCR (Figures 4B–E). To distinguish ectopic *OsATG8a* from the endogenous transcript, two pairs of primers were used for *OsATG8a*, with one targeting the coding sequence and the other targeting the 3’-UTR (Figure 4B). Consistent with the protein levels (Figure 4A), when expressed alone, the *GFP* transcript can accumulate to high levels if driven by 35S or ubiquitin promoters (Figure 4C). In contrast, when expressed as *GFP-OsATG8a*, the transcript level of *GFP*, representing the fusion gene, was clearly repressed (Figure 4C), indicative of transcriptional regulation. *OsATG8a* transcripts, representing both endogenous and ectopic *OsATG8a*, accumulated in all *GFP-ATG8s* lines carrying the three constructs, however not very differently except for one line (Figure 4D), yet again suggesting a regulation at the transcription level. Judging from the levels of the *OsATG8a-UTR*, the endogenous expression of *OsATG8a* was not very different in all lines including the wild type, suggesting that it is the transcription of the fusion gene that gets regulated (Figure 4E). Therefore, both transcriptional and post-transcriptional regulation of ATG8a have taken place in the transgenic lines carrying *GFP-ATG8a*, especially in *Pro35S:GFP-OsATG8a* and *ProUBQ10:GFP-OsATG8a*, resulting in comparable *GFP-OsATG8a* protein levels. The mechanism is currently unknown and awaits further study.

### Immunoprecipitation-Mass Spectrometry Analyses on the Three *GFP-OsATG8a* Transgenic Lines

ATG8 is a star molecule in proteomic studies of autophagy. To see if the *GFP-OsATG8a* transgenic lines could be used for protein–protein interaction studies, GFP-Trap immunoprecipitation was performed using seedlings from three transgenic lines along with GFP control plants grown under control conditions (mock = Hoagland’s solution) or treated with Hoagland’s solution containing 150 mM NaCl for 1 h. The immuno-precipitated proteins were verified with SDS-PAGE and sent for mass spectrometry analyses.

**Figure 5:**
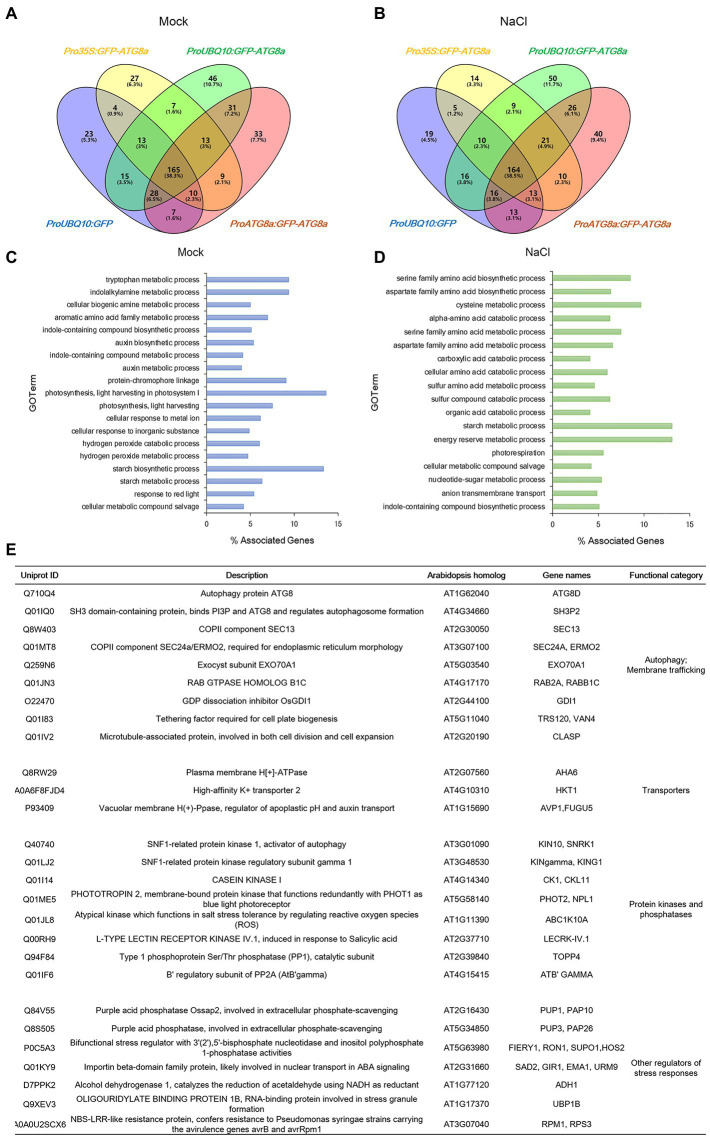
Identification of new ATG8-interacting proteins with immunoprecipitation-mass spectrometry analyses on GFP-OsATG8a marker lines. **(A)** Venn diagram showing the overlap in the number of proteins identified from three GFP-OsATG8a lines and the GFP control line, under normal growth condition (Mock). **(B)** Venn diagram showing the overlap in the number of proteins identified from three *GFP-OsATG8a* lines and the *GFP* control line, treated with 150 mM NaCl for 1 h (NaCl). **(C)** Gene Ontology (GO)-Biological Process (BP) enriched in all OsATG8a-interacting proteins (166 in total) identified from the three transgenic lines under normal growth condition (Mock). **(D)** GO-BP enriched in all 170 OsATG8a-interacting proteins identified after salt stress (NaCl). **(E)** Selected OsATG8a-interacting proteins, including regulators of autophagy and other trafficking routes, plasma membrane and tonoplast transporters, protein kinases and phosphatases, and other regulators of stress responses.

From the eight conditions, 2,244 proteins from 8,120 peptides altogether were obtained. After subtracting the proteins identified in GFP control, 56, 97, and 86 proteins remained for *Pro35S:GFP-OsATG8a*, *ProUBQ10:GFP-OsATG8a*, and *ProOsATG8a:GFP-OsATG8a* under control conditions (Figure 5A). With salt treatment, 54, 106, and 97 proteins were identified as interacting partners for OsATG8a in the three lines, respectively (Figure 5B). The numbers of shared target proteins between different transgenic lines were also shown in the Venn diagrams (Figures 5A,B). To better describe the OsATG8a-interacting protein landscape, we mapped the proteins to the Arabidopsis proteome by batch BLAST and used the Arabidopsis protein with the lowest e-value to represent the rice protein. Then, we combined all target proteins identified from mock or NaCl treatment for Gene Ontology (GO) analysis using Cytoscape as described (Wu et al., 2022). Under controlled (mock) conditions, most enriched GO terms are tryptophan metabolic processes, indolalkylamine metabolic processes, photosynthesis, and starch biosynthesis, indicative of growth-related processes like auxin biosynthesis and photosynthesis (Figure 5C). Such categorization is consistent with the knowledge that basal level autophagy constitutively recycles damaged and obsolete proteins. With salt stress, GO terms related to the amino acid metabolic processes, especially serine, aspartate, and cysteine metabolic processes overwhelmingly enriched (Figure 5D). Energy reserve and starch metabolic processes were also enriched (Figure 5D).

We also randomly generated a list of cytoplasmic proteins that have not been reported to interact with ATG8 (Supplementary Table S4) and searched their protein sequences to see whether they have LIR/AIM. All selected proteins indeed have one or more LIR/AIM, suggesting that they may be ATG8-interating partners.

Apart from the GO analysis, we manually examined the list of potential ATG8-interacting proteins and spotted some very interesting proteins. A manually annotated list is shown in Figure 5E. First of all, like all other ATG8/LC3 interactomes, essential membrane trafficking regulators were identified. These include SH3P2, the ATG8-, and PI3P-binding protein that regulates autophagosome formation; the COPII coat proteins SEC13 and SEC24—COPII vesicles have been revealed to participate in autophagosome formation; EXO70A1, the Exocyst component that had been reported to regulate root growth; Rab2, the Rab GTPase whose mammalian homolog have been reported to regulate autophagy; the Guanosine nucleotide diphosphate dissociation inhibitor GDI1, which is general to Rab GTPases and ROP GTPases; TRS120, the TRAPPII component that is required for cell plate formation; and CLASP, the microtubule-associated protein that regulates both cell division and cell expansion. Plasma membrane- and tonoplast-localized proteins, such as the P-ATPase AHA6, the sodium transporter HKT1, and the Vacuolar H^+^ -Pyrophosphatase AVP1, were also identified. Furthermore, kinase and phosphatases that have been reported to regulate autophagy or stress response were seen in the list. These include the catalytic and regulatory subunits of the plant AMPK, SnRK1/KIN10, the key regulator in autophagy, response to carbon limitation, and circadian clock; Casein kinase 1, an ancient protein kinase that had been reported to regulate selective autophagy; PHOT2, a key blue light receptor and kinase that regulate response to high light; stress responsive kinases ABC1K10A and LECRK-IV.1; TOPP4, an evolutionarily conserved PP1 phosphatase that regulates plant growth and immune response; and ATB’GAMMA, a regulatory subunit of the PP2A phosphatase that is known to balance growth and defense. Other stress response proteins identified include UBP1B, a marker protein for stress granules that stores mRNA during stress conditions, as well as FIERY1/HOS2, SAD2, ADH1, RPM1/RPS3, and PUP1 and PUP3, two purple acid phosphatases that function in response to low phosphate. We concluded that the potential OsATG8a-interacting proteins are worthy of further exploration, and the transgenic lines that we generated can be used by the community in future to identify new OsATG8a-interacting proteins under various developmental and stress conditions.

## Discussion

In this study, we validated that transgenic lines carrying *GFP-OsATG8a*, either driven by the *35S*, the *ubiquitin*, or the endogenous promoter, are suitable for monitoring autophagy in rice. An accidental yet interesting finding is that transcriptional and post-transcriptional regulation occurred when *GFP-OsATG8a* is ectopically expressed in rice (Figure 4). Firstly, both the *35S* and the *ubiquitin* promoter can strongly and effectively drive the expression of GFP. However, the expression levels of *GFP-ATG8a*, driven by the three promoters, are more or less comparable. We analyzed approximately a hundred lines carrying the three constructs with immunoblotting, yet a *GFP-ATG8a* line with the level of GFP-ATG8a comparable to GFP driven by *35S* or *ubiquitin* promoters was not found. For this reason, the promoter selection for the rice GFP-ATG8 marker line may not be as important as for the Arabidopsis GFP-ATG8 markers. It is likely that over-accumulation of the ATG8a protein could exert an adverse effect on reproduction and that a feed-back regulation likely exists for *ATG8a* to maintain a not-too-high level of the ATG8a protein. The detailed mechanism remains to be discovered. The transcription factors that may regulate the expression of *OsATG8a* remains unidentified; checking the rice homologs of the relevant Arabidopsis transcription factors may help to answer the question. A few transcription factors were identified from the IP-MS, and they are also worthy of further examination. Another possibility is through a miRNA that targets ATG8a. In animals, for instance, MIR204 and MIR33 have been reported to target LC3B to suppress autophagy; MIR143 and MIR133A-3p have been reported to target GABARAPL1 to induce autophagy, and MIR195 targets GABARAPL1 to repress autophagy (Akkoc and Gozuacik, 2020). Whether similar mechanism may exist in rice remains to be discovered.

The transcriptional and post-transcriptional regulation on ATG8a could also explain why the three GFP-Trap/Mass Spectrometry experiments generated similar lists of proteins (Figure 5). Repeated identification of the same proteins actually added more confidence to the IP-MS study. A number of proteins identified are worthy of further study and may help answer some existing questions in plant autophagy, such as which membranes or vesicles, apart from COPII vesicles (Zeng et al., 2021; Kim et al., 2022), may contribute to autophagosome formation. Early studies for plant autophagy had identified ATI1 and ATI2 as plant-specific ATG8-interacting proteins; both were uncovered through an Y2H screen (Honig et al., 2012). Both ATIs have recently been characterized as starvation-induced ER-phagy receptors for MSBP1 (Wu et al., 2021). We have recently reported a non-autophagy role for ScATG8 in promoting the degradation of vacuolar membrane proteins when the yeast cells approach the stationary phase (He et al., 2021). A similar process had previously been reported for the fission yeast (Liu et al., 2018), suggesting non-autophagy functions of ATG8 could be widespread. Interestingly, in this process, the vacuolar membrane protein that recruits ATG8 to the vicinity of tonoplast, Hfl1, was homologs to lazarus 1(LAZ1) of Arabidopsis, which was initially identified as a suppressor of *acd11*-related cell death (Malinovsky et al., 2010). The transgenic lines generated in this study, with other screening methods, can be used to identify new OsATG8a-interacting proteins and even non-autophagy functions of plant ATG8s. We hope these rice autophagy marker lines will facilitate both the research and the applications of plant autophagy.

## Data Availability Statement

The original contributions presented in the study are included in the article/Supplementary Material, and further inquiries can be directed to the corresponding authors.

## Author Contributions

QG: conceptualization and writing—review and editing. RL, RZ, and QG: methodology. RL and YY: investigation. RL and QG: writing—original draft. XL and QG: funding acquisition. RZ, XL, and QG: supervision. All authors contributed to the article and approved the submitted version.

## Funding

This work is supported by the National Natural Science Foundation of China (91954102 and 31871355) to QG.

## Conflict of Interest

The authors declare that the research was conducted in the absence of any commercial or financial relationships that could be construed as a potential conflict of interest.

## Publisher’s Note

All claims expressed in this article are solely those of the authors and do not necessarily represent those of their affiliated organizations, or those of the publisher, the editors and the reviewers. Any product that may be evaluated in this article, or claim that may be made by its manufacturer, is not guaranteed or endorsed by the publisher.

## Footnotes

^1^ https://imagej.nih.gov/

## Supplementary Material

Supplementary Figure S1 Visualization of accumulation of autophagic bodies in rice protoplasts. Rice protoplasts expressing *GFP-OsATG8a* driven by **(A)** *UBQ10* promoter, **(B)** *OsATG8a* promoter was observed with LSCM after treatment with 200 nM AZD8055 or 1 μM ConA, or AZD8055 plus ConA, for 3 h. Bar = 7.5 μm.

Supplementary Figure S2 Verification of *GFP* and *GFP-OsATG8a* transgenic lines. T1 transgenic lines regenerated from the transformed rice calli were verified with **(A)** genomic PCR and **(B)** immunoblotting. GFP antibody recognizes GFP-ATG8 bands (40 kD) and free GFP (27 kD). **(C)** GFP-OsATG8a and GFP-OsATG8d from rice transgenic lines (P for plant) or prokaryotically expressed (E for *E.coli*) were detected with a GFP antibody. Primers used in **(A)** are listed in Supplementary Table S1. Anti-histone H3 was used as an internal control in **(B)**.

Supplementary Figure S3 Growth parameters of rice transgenic lines carrying *GFP-OsATG8a*. **(A,B)** T1 transgenic rice plants grown in the paddy field were measured before harvesting. **(A)** Plant height and **(B)** tiller number were measured. Data are means ± SD (*n* = 7–15), one-way ANOVA followed by a Duncan test; *p* = 0.05. Different letters denote significant differences. **(C)** Phenotypes of 16-day-old transgenic rice seedlings. Three T3 lines for each construct were shown. Bar = 3 cm.

## Abbreviations

ATG: Autophagy-related
ConA: Concanamycin A
ER: Endoplasmic reticulum
GFP: Green fluorescent protein
LSCM: Laser Scanning Confocal Microscopy
WT: Wild type
