# Supplementary figures and images for: Monitoring Autophagy in Rice With GFP-ATG8 Marker Lines
Source: Front Plant Sci. 2022 Apr 25;13:866367. doi: 10.3389/fpls.2022.866367 (PMC9083259; doi:10.3389/fpls.2022.866367)

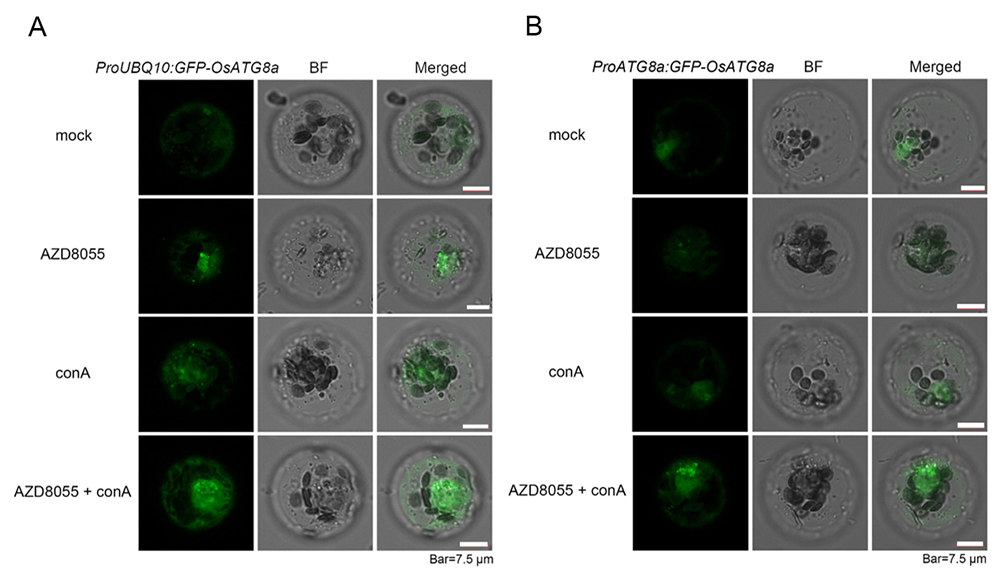

Supplement: Supplementary Figure S1 — Visualization of accumulation of autophagic bodies in rice protoplasts. Rice protoplasts expressing GFP-OsATG8a driven by (A) UBQ10 promoter, (B) OsATG8a promoter was observed with LSCM after treatment with 200 nM AZD8055 or 1 μM ConA, or AZD8055 plus ConA, for 3 h. Bar = 7.5 μm. [file Image_1.TIF]

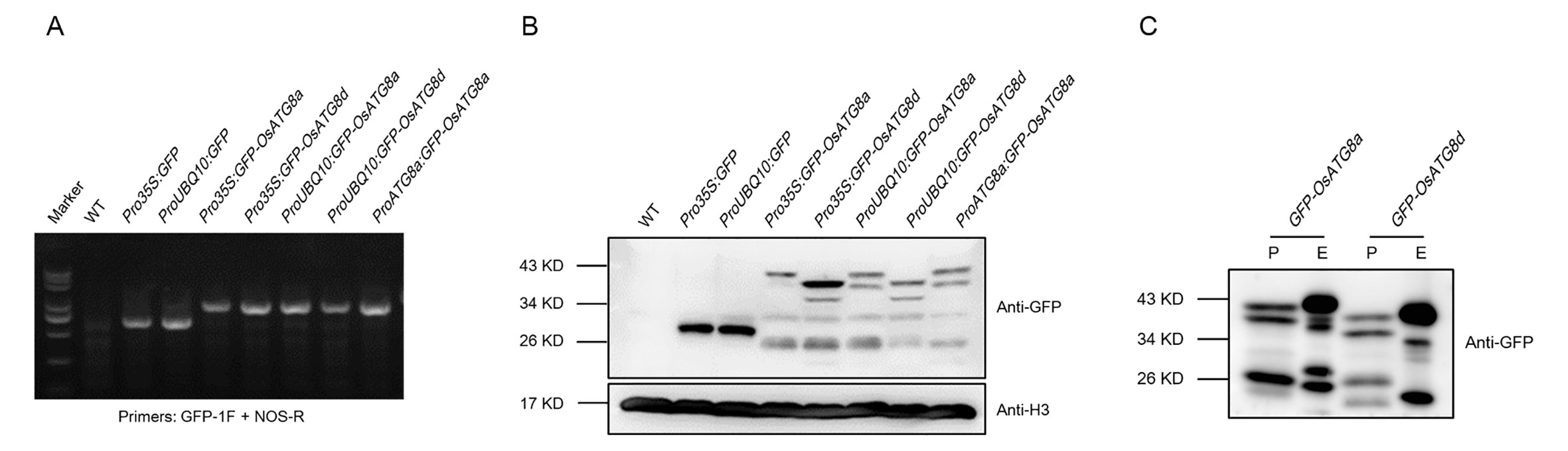

Supplement: Supplementary Figure S2 — Verification of GFP and GFP-OsATG8a transgenic lines. T1 transgenic lines regenerated from the transformed rice calli were verified with (A) genomic PCR and (B) immunoblotting. GFP antibody recognizes GFP-ATG8 bands (40 kD) and free GFP (27 kD). (C) GFP-OsATG8a and GFP-OsATG8d from rice transgenic lines (P for plant) or prokaryotically expressed (E for E.coli) were detected with a GFP antibody. Primers used in (A) are listed in Supplementary Table S1. Anti-histone H3 was used as an internal control in (B). [file Image_2.TIF]

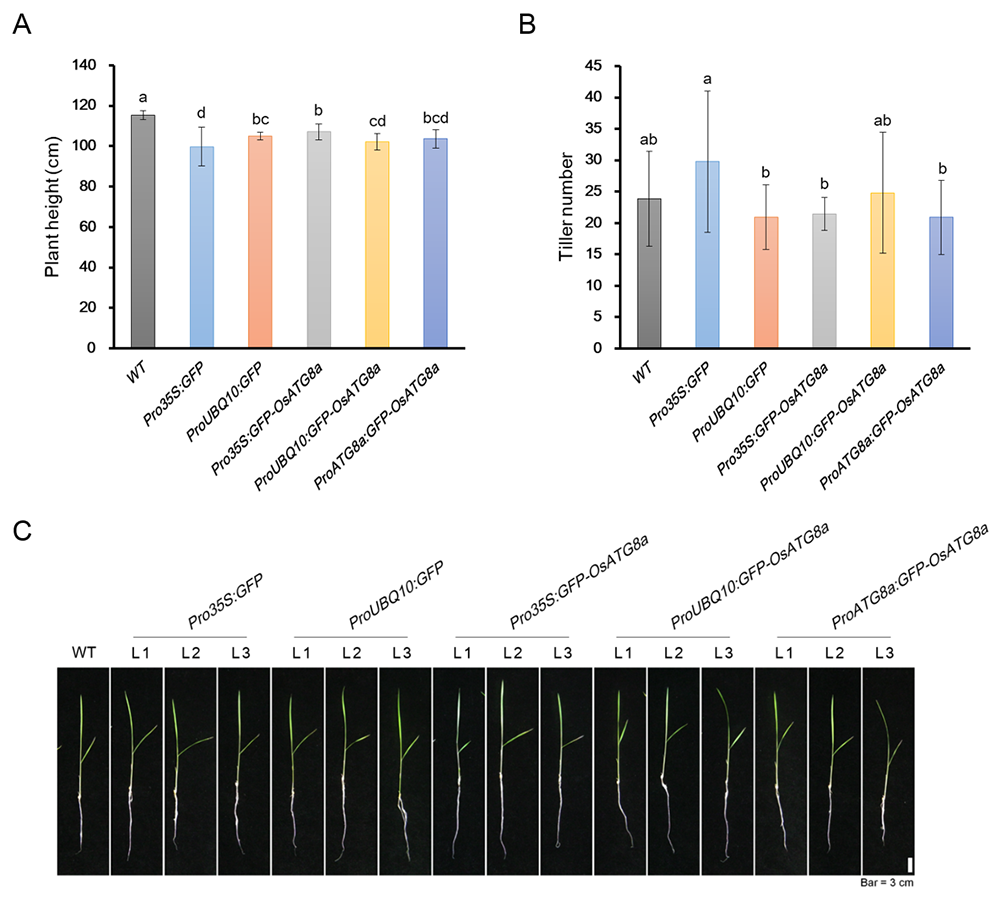

Supplement: Supplementary Figure S3 — Growth parameters of rice transgenic lines carrying GFP-OsATG8a. (A,B) T1 transgenic rice plants grown in the paddy field were measured before harvesting. (A) Plant height and (B) tiller number were measured. Data are means ± SD (n = 7–15), one-way ANOVA followed by a Duncan test; p = 0.05. Different letters denote significant differences. (C) Phenotypes of 16-day-old transgenic rice seedlings. Three T3 lines for each construct were shown. Bar = 3 cm. [file Image_3.TIF]
