# Supplementary material for: Monitoring Autophagy in Rice With GFP-ATG8 Marker Lines
Source: Front Plant Sci. 2022 Apr 25;13:866367. doi: 10.3389/fpls.2022.866367 (PMC9083259; doi:10.3389/fpls.2022.866367)
Supplement: Supplementary file 4 [file Data_Sheet_1.docx]

Supplemental Table S1. Primers used in this study.

Primers for vector construction

| Name | Primer sequences |
| --- | --- |
| ATG8a-F | CACCACCACCACGTGATGGCCAGGACTTCCTTC |
| ATG8a-R | GGGGAAATTCGAGCTGTTACGCAGAGCCGAATGTG |
| ProATG8a-F | CGGGGATCCTCTAGAGTTCGACGAAGTCGGCCCAGCTAGAAC |
| ProATG8a-R | TACTAGTCAGATCTACCATGGCTCCAACCTGCGAATCAAATC |
| GFP-1F | CTACCTGTTCCGTGGCCAAC |
| NOS-R | TAATCATCGCAAGACCGGCA |

Primers for PCR or qRT-PCR

| Name | Primer sequences |
| --- | --- |
| GFP-1F | CTACCTGTTCCGTGGCCAAC |
| NOS-R | TAATCATCGCAAGACCGGCA |
| rEF-F | CTGCAACAAGATGGATGCCA |
| rEF-1R | GTAGCCGACCTTCTTCAGGT |
| GFP-F | ACCTGTCCACACAATCTGCC |
| rGFP-R | ATGCCATGTGTAATCCCAGC |
| rATG8a-1F | CCAGTTTGTCTATGTGGTTCGG |
| rATG8a-R | ATCAAAGAAGCAGTTGGCGG |
| rATG8a-UTR-F | CATCAACTGTTGCTGCTGCTG |
| rATG8a-UTR-R | CCATTGAGGCAATCCAGCACAG |

Supplemental Table S2. Shared interacting proteins for OsATG8a in the three marker lines.

| Accession | Description |
| --- | --- |
| A0A0F7EX29 | Ribulose bisphosphate carboxylase large chain (Fragment) OS=Oryza sativa OX=4530 GN=rbcL PE=3 SV=1 |
| Q36688 | Ribulose bisphosphate carboxylase small chain OS=Oryza sativa OX=4530 GN=rbcS PE=2 SV=1 |
| Q01H94 | B0103C08-B0602B01.9 protein OS=Oryza sativa OX=4530 GN=B0103C08-B0602B01.9 PE=3 SV=1 |
| Q259D2 | NADPH-protochlorophyllide oxidoreductase OS=Oryza sativa OX=4530 GN=H0801D08.7 PE=3 SV=1 |
| Q8S870 | Putative class III chitinase OS=Oryza sativa OX=4530 GN=OSJNBa0061K21.7 PE=3 SV=1 |
| A0A482DUT2 | Peroxidase OS=Oryza sativa OX=4530 PE=3 SV=1 |
| Q8S505 | Purple acid phosphatase OS=Oryza sativa OX=4530 PE=2 SV=1 |
| Q259D7 | H0801D08.15 protein OS=Oryza sativa OX=4530 GN=H0801D08.15 PE=4 SV=1 |
| Q8LPC9 | Threonyl-tRNA synthetase (Fragment) OS=Oryza sativa OX=4530 GN=ThrRS PE=2 SV=1 |
| Q8S3J6 | Putative dihydroorotate dehydrogenase (Fragment) OS=Oryza sativa OX=4530 PE=2 SV=1 |
| Q01JD7 | OSIGBa0130P02.2 protein OS=Oryza sativa OX=4530 GN=OSIGBa0130P02.2 PE=4 SV=1 |
| O04978 | Ascorbate oxidase (Fragment) OS=Oryza sativa OX=4530 PE=2 SV=1 |
| J9UPY5 | Anthranilate synthase OS=Oryza sativa OX=4530 GN=OASA1 PE=3 SV=1 |

Supplemental Table S3. Shared interacting proteins for OsATG8a in the three marker lines with NaCl treatment.

| Accession | Description |
| --- | --- |
| Q710Q4 | Autophagy-related protein (Fragment), OsATG8 OS=Oryza sativa OX=4530 GN=xd2 PE=2 SV=1 |
| O22487 | Ribulose bisphosphate carboxylase small chain OS=Oryza sativa OX=4530 PE=2 SV=1 |
| A0A0R7FMX6 | Catalase OS=Oryza sativa OX=4530 PE=2 SV=1 |
| Q7M1Y7 | Photosystem II oxygen-evolving complex protein 2 (Fragment) OS=Oryza sativa OX=4530 PE=1 SV=1 |
| Q01HY2 | OSIGBa0138H21-OSIGBa0138E01.14 protein OS=Oryza sativa OX=4530 GN=OSIGBa0138H21-OSIGBa0138E01.14 PE=3 SV=1 |
| P0C5A3 | 3'(2'),5'-bisphosphate nucleotidase OS=Oryza sativa OX=4530 PE=2 SV=1 |
| Q01IV2 | OSIGBa0157A06.7 protein OS=Oryza sativa OX=4530 GN=OSIGBa0157A06.7 PE=4 SV=1 |
| Q9M3W1 | Putative Bowman Birk trypsin inhibitor OS=Oryza sativa OX=4530 GN=rbbi2-3 PE=3 SV=1 |
| Q01JQ5 | H0523F07.8 protein OS=Oryza sativa OX=4530 GN=H0523F07.8 PE=4 SV=1 |
| Q710Q5 | H+-transporting ATP synthase (Fragment) OS=Oryza sativa OX=4530 GN=xd1 PE=2 SV=1 |
| Q258Y7 | H0624F09.11 protein OS=Oryza sativa OX=4530 GN=H0624F09.11 PE=3 SV=1 |
| Q01L44 | H0502B11.7 protein OS=Oryza sativa OX=4530 GN=H0502B11.7 PE=3 SV=1 |
| Q01K86 | OSIGBa0148A10.12 protein OS=Oryza sativa OX=4530 GN=OSIGBa0148A10.12 PE=3 SV=1 |
| Q8RYA7 | Drought-induced S-like ribonuclease OS=Oryza sativa OX=4530 PE=2 SV=1 |
| A0A0B4U0L7 | Dehydrogenase (Fragment) OS=Oryza sativa OX=4530 GN=LOC_Os01g53910.1 PE=2 SV=1 |
| Q01KT2 | OSIGBa0140A01.7 protein OS=Oryza sativa OX=4530 GN=OSIGBa0140A01.7 PE=4 SV=1 |
| D5L625 | CIN1 (Fragment) OS=Oryza sativa OX=4530 GN=cin1 PE=3 SV=2 |
| Q9ZSS9 | 50S ribosomal protein L10 OS=Oryza sativa OX=4530 GN=rpl10 PE=2 SV=1 |
| A0A411LC64 | Cytochrome c oxidase subunit 2 OS=Oryza sativa OX=4530 GN=cox2 PE=3 SV=1 |
| Q01J73 | H0818E04.5 protein OS=Oryza sativa OX=4530 GN=OSIGBa0152K17.14 PE=3 SV=1 |
| Q01I61 | H0315A08.11 protein OS=Oryza sativa OX=4530 GN=H0315A08.11 PE=3 SV=1 |

Supplemental Table S4. Analysis of potential interacting proteins for OsATG8a.

| **Accession** | **Description** | **Domain** | | | **Location** | **Locus** | **Gene names** |
| --- | --- | --- | --- | --- | --- | --- | --- |
| Q01HY2 | OSIGBa0138H21-OSIGBa0138E01.14 protein | Glyco_hydro_3_C, Glyco_hydro_3, Fn3-like | AIM |  | Extracellular region | AT5G64570 | ATBXL4, XYL4 |
| P0C5A3 | 3'(2'),5'-bisphosphate nucleotidase | Inositol_P | AIM |  | Nucleus or Cytoplasm | AT5G63980 | ALX8, ATFRY1, ATSAL1, FIERY1, FRY1, HOS2, RON1, ROTUNDA 1 |
| Q01IV2 | OSIGBa0157A06.7 protein | CLASP_N | AIM | UIM | Cytoplasm | AT2G20190 | ATCLASP, CLASP |
| Q9M3W1 | Putative Bowman Birk trypsin inhibitor | Bowman-Birk_leg, Bowman-Birk_leg, Bowman-Birk_leg | AIM |  | Extracellular region |  |  |
| Q258Y7 | H0624F09.11 protein | DJ-1_Pfpl, DJ-1_Pfpl | AIM | UIM | Cytoplasm | AT3G02720 | DJ-1D, DJ1D |
| Q8RYA7 | Drought-induced S-like ribonuclease | Ribonuclease_T2 | AIM |  | Extracellular region | AT1G26820 | RIBONUCLEASE 3, RNS3 |
| Q01KT2 | OSIGBa0140A01.7 protein | NTF2, RRM_1 | AIM | UIM | Nucleus or Cytoplasm | AT5G60980 | NTF2 family protein with RNA binding domain-containing protein |
| Q01I61 | H0315A08.11 protein | DUF150 | AIM |  | Cytoplasm | AT1G77122 | Uncharacterized protein family UPF0090 |
